# A Balanced Chromosomal Rearrangement Disrupting the Telomerase RNA Component Gene Causes Short Telomere-Mediated Pulmonary Fibrosis

**DOI:** 10.3390/ijms27198667

**Published:** 2026-09-28

**Authors:** Noa Hourvitz, Monica Neustadter-Blackman, Avraham Unterman, Riham Smoom, Adi Mory, Hila Lederman Nahemias, Alina Kurolap, Uri Hamiel, Mordechai R. Kramer, Hagit Baris Feldman, Yehuda Tzfati, Daphna Marom

**Affiliations:** 1 Department of Genetics, The Silberman Institute for Life Sciences, Safra Campus, The Hebrew University of Jerusalem, Jerusalem 91904, Israel; noa.hourvitz@mail.huji.ac.il (N.H.); riham.samman@mail.huji.ac.il (R.S.); 2 The Genetic Institute and Genomics Center, Tel Aviv Sourasky University Medical Center, Tel Aviv 6423906, Israelhilana@tlvmc.gov.il (H.L.N.);; 3 Pulmonary Fibrosis Center of Excellence, Tel Aviv Sourasky University Medical Center, Tel Aviv 6423906, Israel; 4 Gray Faculty of Medical & Health Sciences, Tel Aviv University, Tel Aviv P.O. Box 39040, Israel; 5 Pulmonary Institute, Rabin Medical Center, Petah-Tikva 4941492, Israel

**Keywords:** telomere biology disorders, long-read nanopore sequencing, telomerase, *TERC*, balanced translocation

## Abstract

Excessive telomere shortening contributes to pulmonary fibrosis (PF), with at least 30% of familial cases attributed to telomere biology disorders (TBDs). However, TBD-associated PF remains underdiagnosed because of unidentified disease-causing mutations and limited referral for telomere and genetic testing. Here, we combined telomeric restriction fragment analysis, telomere analysis by a long-read Nanopore sequencing method (NanoTelSeq), exome sequencing (ES), and optical genome mapping (OGM) to investigate the underlying cause of disease in a family affected by PF, microcephaly, and premature hair graying. We identified a familial balanced translocation associated with short telomeres, which involved a chromosome 3 breakpoint within the non-coding telomerase RNA component (*TERC*) gene, disrupting the 451-nucleotide telomerase RNA. Establishing the diagnosis enabled a TBD-tailored protocol and successful lung transplantation in the proband at age 49. These findings identified structural disruption of *TERC* as a previously unrecognized cause of TBD and broadened the genetic landscape of TBD beyond splicing, single nucleotide substitutions, and insertion/deletion variants. Furthermore, they demonatrate the importance of routine telomere length measurement alongside comprehensive genomic testing in familial PF.

## 1. Introduction

Telomere biology disorders (TBDs), a group of rare heterogeneous diseases, are caused by germline variants in genes important for telomere maintenance and characterized by abnormally short or dysfunctional telomeres [1]. The most common manifestation of adult-onset TBD is pulmonary fibrosis (PF), which typically presents at a younger age and follows a more progressive disease course than non-TBD PF [1,2].

Up to 30% of familial PF (FPF) and 10% of sporadic PF cases are associated with pathogenic variants in single genes, most commonly in telomerase or other telomere maintenance genes, such as *TERT*, *RTEL1*, *PARN*, and *TERC* [3,4]. Variants are mostly missense, but nonsense, frameshift and splice-site variants have also been reported [1]. Monogenic TBD-related PF typically shows autosomal dominant inheritance with reduced penetrance and inter- or intra-familial variable expressivity. Anticipation, driven by progressive telomere shortening across successive generations with the same TBD-causing variant, has also been described [5].

Currently, there is no cure for TBD-related PF other than lung transplantation, which is reported to be more frequent in these patients than in non-TBD patients [6]. A TBD diagnosis has important management implications, given the high sensitivity of TBD patients to anti-inflammatory immunosuppressive agents and the need for tailored peri-transplant protocols [7]. In addition, TBD diagnosis affects familial surveillance and possibly family planning. Therefore, it is crucial to diagnose TBD-related PF in a timely manner.

The diagnosis of TBD relies on the demonstration of short telomere length (TL) in peripheral blood leukocytes (PBLs) complemented by next-generation sequencing (NGS) of known TBD genes [8,9]. TL below the 1st percentile is considered diagnostic of TBD in the appropriate clinical setting, while TL below the 10th percentile is an accepted screening threshold suggestive of adult-onset TBD. However, PF patients are not routinely screened for TL or for genetic variants [10,11]. The standard clinical test, flow-FISH, measures average TL, presented as age-adjusted percentiles [12]. Telomeric restriction fragment (TRF) analysis, the gold-standard method in the research setting, provides both the average TL and a visual image of its distribution. However, neither method measures the length or fraction of the shortest telomeres. This specific subset is considered the most biologically significant subset, as it affects cellular physiology and drives disease [13,14]. Consequently, patients with an average TL that falls within the normal range, or those with short telomeres below the 10th percentile but no identifiable TBD-causing variant, pose diagnostic challenges in TBD-related PF [15]. To address these limitations, we used NanoTelSeq, which measures single telomeres at high accuracy, including the shortest telomeres [16].

We describe a unique family affected by PF, harboring a novel reciprocal translocation that disrupts the non-coding RNA gene *TERC*. We identified and confirmed this disruption using multiple complementary technologies. Telomere analysis by NanoTelSeq revealed severely short telomeres in the proband, over 15% of which were shorter than 2 kb. Family member evaluation showed variable phenotypic expressivity, with short telomere length co-segregating with the chromosomal translocation, along with possible phenocopy and anticipation. Clinical course, phenotypic features and diagnostic approaches are discussed.

## 2. Results

### 2.1. Clinical Course of Pulmonary Disease

The proband presented at age 40 with exertional dyspnea and a family history of PF (mother). She had a minimal smoking history, no relevant occupational exposures, and a remote episode of self-limited arthritis of unclear etiology. Examination showed clubbing and minimal bibasilar “velcro” crackles, without clinical features of connective-tissue disease. Resting oxygen saturation was normal. Baseline forced vital capacity (FVC) was 65% predicted, and diffusion capacity for carbon monoxide (DLCO) was 39%. Chest computed tomography (CT) showed upper-lobe pleuroparenchymal fibroelastosis (PPFE) and a lower-lobe indeterminate usual interstitial pneumonia (UIP) pattern. Bronchoscopy was non-diagnostic, demonstrating mild septal fibrosis and few germinal centers; BAL showed 3% neutrophils, 12% eosinophils, 7% lymphocytes, and 77% macrophages.

In accordance with a working diagnosis of idiopathic pulmonary fibrosis (IPF), antifibrotic therapy was initiated with nintedanib, which was replaced by pirfenidone due to intolerance to the former. Despite treatment, she developed progressive radiologic fibrosis (Figure 1A) and physiologic decline. Seven years after presentation, her diagnosis was reconsidered because of mold and bird exposure, elevated inflammatory markers, prior arthritis, and germinal centers on biopsy. However, nailfold capillaroscopy and broad autoimmune serologies were negative, and no other connective-tissue disease manifestations were present. A provisional diagnosis of fibrotic hypersensitivity pneumonitis (fHP) initiated prednisone treatment, which was later discontinued due to disease progression.

By age 49, she required continuous oxygen, her FVC had declined to 28%, her DLCO was unmeasurable, and her 6 min walk test was 278 m on oxygen, compared with 480 m on room air three years earlier. She underwent successful lung transplantation nine years after presentation. Explant pathology showed predominantly peripheral interstitial fibrosis with focal peripheral sparing, peribronchiolar fibrosis, patchy chronic inflammation, and a rare possible non-necrotizing granuloma, supporting fHP. At 1.5 years post-transplantation, after one steroid-responsive rejection episode, she is clinically stable with near-normal lung function (for additional clinical data, see Supplementary Materials).

### 2.2. Genetic Counseling and Phenotypic Features

The proband was referred to genetic counseling at the age of 47 years. Personal history and physical examination revealed microcephaly (−2.7 SD), premature hair graying since the age of 11 years, frontal fibrosing alopecia, and osteoporosis. The proband’s mother (I1, Figure 1B) was diagnosed with IPF at the age of 74 years and passed away two years later due to PF complications. Both of the proband’s daughters (III1, III2) were born with bilateral isolated congenital cataracts, surgically removed in childhood. The eldest (III1) was a healthy 19-year-old at the time of referral; her physical examination was normal. The younger daughter (III2) was 12-year-old at the time of referral. During the proband’s pregnancy with her, a ventricular septal defect (VSD) was detected, prompting prenatal karyotyping of amniocytes. The karyotype revealed a balanced reciprocal translocation 46,XX,t(1;3)(q24;q23) (Figure 1C). Physical examination at referral revealed microcephaly (−2 SD) and several gray hairs. Her VSD was hemodynamically insignificant. The proband’s 65-year-old sister (II3) reported premature hair graying and heavy smoking at time of referral. Her physical examination was unremarkable. Her head circumference was 53.5 cm (22nd percentile) and height was 169 cm (81st percentile). The proband’s niece (III3) was reported to have premature hair graying and chronic respiratory symptoms but declined further evaluation and consultation.

Familial karyotyping revealed that the proband, her sister, and the proband’s younger daughter (II1, II3 and III2) harbored the same balanced 46,XX,t(1;3)(q24;q23) translocation, which was interpreted at the time as benign (Figure 1C). The proband’s eldest daughter (III1) did not harbor the translocation and her karyotype was normal. Other family members were either not karyotyped or their reports were unavailable.

### 2.3. Telomere Length

The proband’s physical features and family history raised a strong suspicion of TBD-related FPF, warranting TL measurement. Flow-FISH was not performed due to a lack of public healthcare coverage. Instead, TRF was analyzed in peripheral blood leukocyte samples obtained from the proband (II1), her daughters (III1 and III2), and her sister (II3) by in-gel hybridization to a telomeric probe (Figure 2A, Table 1). The proband’s mean TRF length calculated by WALTER [17] was markedly shorter than that of an age-matched control (3.4 and 6.4 kb, respectively). Her sister (II3) displayed a similarly short mean TRF length (3.5 kb). Both daughters, III1 and III2, appeared to have a shorter TRF length than that of the 55-year-old control. However, they demonstrated different results using two computational tools (WALTER [17] and TeloTool [18]): for daughter III1, the mean TRF length was 5.1 kb when calculated by WALTER and 8.2 kb by TeloTool, and for daughter III2, the mean TRF length was 4.6 kb by WALTER and 10.9 kb by TeloTool. These conflicting results reflect a fundamental difference between the two computational processes. TeloTool considers the low-molecular-weight flank of the TRF intensity profile more reliable and reconstructs the high-molecular-weight flank and full profile by Gaussian-fitting. The mean is calculated from the reconstructed distribution and depends on the assumption that the TRF distribution is unimodal and symmetrical. Thus, the corrected profile is consequently symmetric by construction, and TeloTool’s mean and median are identical by definition [18]. WALTER, on the other hand, reports a weighted median of the smoothed raw telomeric signal and makes no distribution assumptions [17]. The calculated values differ between the methods for the daughters and the control but not for the proband and her sister (Figure 2A, Table 1). This discrepancy is consistent with previous comparisons of the two tools, which reported TeloTool overestimation that is most marked for longer telomeres [17,19]. Furthermore, inspection of the TRF profiles shows that they are different between the two daughters III1 and III2, and that III2 does not display a normal distribution (Figure 2A). Daughter III2 also displayed the largest discrepancy between the two methods (10.9 and 4.6 kb for TeloTool and WALTER, respectively), suggesting that the non-normal distribution added to the error. The large differences between the calculated values are sufficient to alter the clinical interpretation, and the lack of consistency compromised the ability to use these test results to aid in diagnosis.

To further analyze TL at higher resolution and accuracy, we used a novel method for single telomere measurement by long-read Nanopore sequencing, termed NanoTelSeq [16]. NanoTelSeq provides accurate, single-telomere length measurements and delivers a representative distribution of telomere lengths, including the shortest ones. Obtaining the accurate sequence enables distinguishing canonical telomeric repeats (TTAGGG) and telomere variant repeats (TVRs), containing one mismatch within the canonical 6-nucleotide repeat unit. TVRs may exhibit a reduced affinity for shelterin proteins or an altered ability to form protective structures [20], potentially compromising telomere protection and function (Supplementary Figure S1A). The inclusion of TVRs in TL measurement (Figure 2B, Table 1) shows that the proband’s median TL was 2.97 kb and 17.7% of the telomeres were shorter than 2 kb, as compared to unrelated healthy female controls, aged 62 yo, 55 yo or 47 yo, with 6.03 kb, 5.85 kb or 6.22 kb median TL and 1.8%, 1.3% or 1.4% below 2 kb, respectively. Considering only the canonical (TTAGGG) repeats and not TVRs, the calculated proband’s median TL was 2.35 kb and 36.7% of the telomeres were below 2 kb, as compared to the 62 yo, 55 yo or 47 yo controls, with 5.56 kb, 5.34 kb or 5.93 kb and 4.7%, 3.4% or 2.9% below 2 kb, respectively (Figure 2C). Analysis of the proband’s telomeric sequences (Supplementary Figure S1B) shows that canonical repeats were progressively lost, while proximal TVRs remained, constituting in some telomeres most of the remaining telomere sequence. The median TL values of the proband’s sister (II3) were similar to those of the proband, 3.10 kb with TVRs and 2.54 kb without TVRs, and much shorter than the controls (Figure 2B,C and Table 1). The percentage of telomeres below 2 kb was 13.7% with TVRs and 32.4% without TVRs, respectively. Regarding the proband’s daughters, NanoTelSeq revealed that the younger daughter (III2) had markedly shorter telomeres than her sister (III1), with a median length of 4.26 kb compared to 5.46 kb (with TVRs) and 3.81 kb compared to 5.03 kb (without TVRs), respectively. However, both sisters had a much shorter median TL than the youngest control 28 yo (7.91 and 7.59 kb, with and without TVRs; Figure 2B,C, Table 1). Most importantly, 8.5% of the telomeres of daughter III2, but only 3.0% of the telomeres of daughter III1, were shorter than 2 kb when including TVRs (19.3 and 7.1% without TVRs), as compared to the control (0.5% and 1.2%, with and without TVRs). The shorter telomeres of the younger daughter, III2, and the higher percentage of short telomeres were consistent with the clinical presentation of premature hair graying and microcephaly and segregated with the translocation found in III2 but not in III1.

### 2.4. Genetic Testing

Initial genetic testing included chromosomal microarray (CMA) analysis for the proband, which was normal, and a PF and TBD gene panel analysis (for the full list of genes, refer to Supplementary Materials), which was negative. We proceeded with clinical short-read exome sequencing (ES) with automatic prioritization of candidate variants; however, this too yielded negative results. Given the reciprocal translocation identified by karyotype as 46,XX,t(1;3)(q24;q23), we manually analyzed the cytogenetic breakpoints on both chromosomes. This analysis revealed misaligned sequences at chromosome 3q26.2 (Chr3:169,482,540–169,482,599, GRCh37/hg19) that partially spanned the *TERC* gene, which in fact mapped to chromosome 1 (Supplementary Figure S2B). Manual inspection of the breakpoint on the long arm of chromosome 1 similarly revealed misaligned reads at 1q32.1 (Chr1:199,639,088–199,639,109, GRCh37/hg19), whose sequence mapped to the *TERC* gene (Supplementary Figure S2A). The same findings were observed in II3 and III2 but not in III1 (Supplementary Figure S3).

Further investigation confirmed that the translocation split the *TERC* gene on chromosome 3q26.2 into two parts (Figure 3B,D). The first, including the 5′ end of the *TERC* gene up to the position corresponding to n.249 of the 451-nucleotide *TERC* RNA sequence, broke from chromosome 3 and fused to chromosome 1. The second, starting at the position corresponding to n.310 of the *TERC* RNA sequence and reaching the 3′ end of *TERC*, remained and was fused to the translocated fragment of chromosome 1 (Figure 3D). A missing sequence of 60 nt in the *TERC* gene was presumably degraded during the repair of the breaks that resulted in the translocation. This focused analysis refined the intragenic disruption breakpoints to Chr1:199,639,088–199,639,109 and Chr3:169,482,540–169,482,599 (GRCh37/hg19), at least 20 Mb downstream of the breakpoints reported by conventional karyotype (Figure 3A,B). Further ES testing of the sister and both daughters confirmed the co-segregation of the *TERC*-disrupting translocation with the clinical presentation, as described above (Table 1, Supplementary Figure S3).

### 2.5. Optical Genome Mapping

To validate the results obtained by ES, we employed optical genome mapping (OGM). This imaging technology examines the fluorescent labeling pattern of individual DNA molecules to identify genome-wide structural variants at a resolution down to 500 base pairs, far exceeding that of conventional cytogenetic approaches. OGM results were consistent with the ES-defined breakpoints and identified the cytogenetic notation to t(1;3)(q32.1;q26.2) (chr1:199,638,595; chr3:169,487,747, GRCh37/hg19) (Figure 3B,C and Figure S4). A summary of the translocation and updated breakage points is illustrated in Figure 3A,B.

## 3. Discussion

We describe a unique familial case of PF caused by *TERC* disruption resulting from a balanced reciprocal translocation. Detecting balanced genomic translocations requires specific diagnostic platforms (summarized in Supplementary Table S1). Traditional G-banded karyotyping and OGM can identify copy-number-neutral rearrangements, whereas automated CMAs and short-read ES, which are usually employed as 1st- and 2nd-tier clinical testing strategies, are usually blind to them. Furthermore, while karyotyping provides global chromosomal context, it is constrained by a low-resolution limit of 5 to 10 megabases. Conversely, OGM offers high-throughput, genome-wide detection down to 0.5 kilobases, narrowing down the actual breakpoint segment but still lacking single-nucleotide breakpoint resolution. Manual targeted split-read review of sequencing data can pinpoint exact breakpoint sequences, but its application is labor-intensive, requires a high index of suspicion and is largely restricted to unique, non-repetitive genomic regions. The translocation described here was initially detected by conventional prenatal karyotyping and classified as benign, with its reported breakage points located away from any known PF or TBD gene. Automated ES analysis did not identify the gene disruption. However, targeted manual review identified split-read discordance within the *TERC* gene, which was localized by OGM to the site of the chromosomal breakpoint.

*TERC* is a 451 nt RNA that includes the template for telomere synthesis by the telomerase reverse transcriptase and additional domains essential for telomerase complex assembly and function [1]. The transcription of the translocated 5′ fragment of the *TERC* gene is expected to generate a transcript that includes the template-pseudoknot domain but not the essential CR4-CR5 domain, the CAB box required for binding of TCAB1, or the Box H/ACA required for binding the Box H/ACA complex and stabilization of the *TERC* RNA (Figure 3D) [1]. The 3′ fragment of the *TERC* gene lacks the promoter, the template-pseudoknot domain and most of the CR4-CR5 domain, and thus it cannot facilitate telomere synthesis. Furthermore, a 60 nt sequence (n.250–309, Figure 3D) is deleted. Altogether, the disruption of the *TERC* gene by the translocation is expected to abolish the telomerase activity of the translocated *TERC* allele and cause telomerase haploinsufficiency. To the best of our knowledge, this is the first report of TBD caused by a balanced chromosomal rearrangement. Notably, this novel *TERC* disruption aligns with a recent study demonstrating that balanced translocations can cause disease through the disruption or dysregulation of non-coding RNAs [21]. This finding reinforces the pathogenic relevance of these genomic elements in hereditary disorders and highlights the importance of accurate breakpoint annotation. Therefore, familial balanced reciprocal translocations, usually interpreted as cytogenetically benign, may in fact disrupt even a short, single-exon gene, such as *TERC*, and cause severe disease. To assist clinicians in their diagnosis of suspected TBD-PF patients we present a diagnostic workflow suggesting simultaneous genetic and TL testing in patients manifesting suggestive features rather than sequential testing (Figure 4).

Our study underscores the limitations of common methods that only estimate mean TL. This is exemplified in the proband’s daughters (III1 and III2; Figure 2, Table 1), where analysis of TRF data using two established computational tools, WALTER and TeloTool, yielded inconsistent results. These discrepancies presumably reflect an abnormal TL distribution and precluded clear identification of which daughter was at risk of developing TBD. In contrast, long-read-sequencing-based NanoTelSeq enabled high-resolution measurement of individual telomeres, allowing accurate estimation of median TL and, importantly, the portion of telomeres shorter than 2 kb. We hypothesize that inclusion of TVRs in TL calculation may lead to overestimation of the effective functional TL (Supplementary Figure S1A,C). NanoTelSeq provides an accurate tool, previously unavailable, to sequence and study the functional and clinical consequences of TVR presence at telomeres in future research.

The 19-year-old daughter (III1), although not carrying the translocation, exhibited shorter median TL (5.46 kb) and a higher percentage of telomeres below 2 kb (3.0%) compared to all unrelated controls (Figure 2B,C and Table 1). Given the estimated average rate of telomere shortening of 43 bp per year in the general population [22], one would expect the telomeres of a 19-year-old individual to be longer, depending on the age difference, rather than shorter than the older controls. Interestingly, both daughters (III1 and III2) were born with bilateral congenital cataracts, and ES did not reveal an alternative shared causative variant. Although not a typical TBD feature, congenital or early-onset cataract has been reported in patients with mutations in *DKC1*, *NOP10* or *NHP2*, which encode three of the four subunits of the Box H/ACA complex that stabilizes telomerase RNA [23,24]. We hypothesize that the short telomeres inherited from their mother, the proband, may have contributed to the development of bilateral congenital cataracts in both daughters. While this hypothesize is not proven, in view of the shorter telomeres in the translocation-negative sister (III1), she may still be at risk for developing TBD-associated ailments in the future, a phenomenon referred to as phenocopy [25,26,27].

Another intriguing finding in this family is the stark phenotypic difference between the proband, who presented with severe PF and additional systemic manifestations, and her older sister (II3), who, despite a history of heavy smoking, was asymptomatic apart from premature hair graying. This observation is consistent with the known contribution of environmental factors and the recently suggested impact of polygenic modifiers [28] to the incomplete penetrance and variable expressivity observed in TBDs, and particularly in PF. However, the physiological basis for such incomplete penetrance among individuals with the same TBD-causing variant remains poorly understood. Whether the lower percentage of critically short telomeres observed in the 18 years older sister (13.7%; with TVRs) compared to the younger proband (17.7%; Figure 2, Table 1) confers a reduced risk of developing disease remains unclear. The relationship between the burden of critically short telomeres and the clinical onset of symptoms requires longitudinal evaluation across different ages and populations, including in the general population, TBD patients, and individuals with TBD-associated variants. Improved risk stratification of individuals with short telomeres is essential for appropriate surveillance and clinical management [7,29]. The proband was ultimately diagnosed with fHP, supporting the current understanding that TBDs predispose to pulmonary fibrosis broadly rather than to a specific clinicopathologic subtype [30].

A limitation of our study is the lack of validated standard TL values measured by NanoTelSeq for the normal population. The controls shown in Figure 2 are examples from the normal population, but they do not represent the entire normal range of telomere lengths, which awaits further characterization. However, the dramatically short TL measured by TRF and NanoTelSeq in the proband and her sister and the segregation of TL with *TERC* disruption clearly support our conclusions. In addition, little is known about the functional consequences of various TVR sequences and whether they should be considered part of the telomere or not, for research and for clinical diagnostic purposes. Therefore, we present the analysis both including and excluding TVRs. Future research will clarify the functional contribution of various TVR sequences and enable the establishment of a more physiologically relevant “effective TL” rather than relying only on the physical TL used today. A further limitation of our study is the lack of direct functional validation of *TERC* disruption as the pathogenic mechanism. We did not assess directly the effects of the translocation on *TERC* RNA expression, transcript integrity, allele-specific expression, or telomerase activity (e.g., by TRAP assay) in patient-derived cells. However, the loss of essential *TERC* domains is expected to abolish telomerase activity. This strongly supports pathogenicity, given that heterozygous loss-of-function and haploinsufficient *TERC* mutations are well established in the literature as a cause of TBD in general and of PF in particular [1,26,31]. Taken together with the clinical phenotypes, the severely short telomeres in the proband, and their segregation with the translocation across affected and unaffected family members, we propose that the disrupted *TERC* caused telomerase dysfunction and TBD-PF. Future functional studies are needed to elucidate the etiological mechanism.

In summary, we present a family with a novel *TERC* disruption secondary to a familial translocation [t(1;3)(q32.1;q26.2), chr1:199,639,088–199,639,109; chr3:169,482,540–169,482,599, *GRCh37/hg19*], causing short telomeres and severe TBD-related PF. Our findings underscore the need for a high index of suspicion and close attention to physical findings and family history suggestive of TBD. We highlight the crucial role of genetic counseling and TL measurement in the evaluation of these patients and in employing complementary genomic technologies to detect a causative variant that may not be identified by clinically available tests, such as CMA and short-read NGS with automated analysis pipelines (Figure 4). Notably, OGM enabled identification of the *TERC* disruption, consistent with manual ES reanalysis. Finally, NanoTelSeq allowed accurate measurement of telomere length, the fraction of the shortest telomeres, and their sequence. However, NanoTelSeq is not yet clinically validated. Using it for clinical diagnosis would require characterizing the normal range of telomere length, sequence variations and abundance of short telomeres in different ages of the general population and in patients. Establishing NanoTelSeq as a diagnostic and prognostic tool would enable optimal TBD management for both patients and at-risk family members.

## 4. Materials and Methods

The study was done in accordance with the Institutional Review Board of the Tel Aviv Sourasky University Medical Center approved protocols (0586-20-TLV, 0276-11-TLV). Informed consent was obtained from participants or parents of minors.

### 4.1. Genetic Studies

Karyotype, chromosomal microarray and virtual (exome-based) panel analysis were performed according to standard protocols. Exome sequencing (ES) and OGM were performed at The Genetics Institute and Genomics Center, Tel Aviv Sourasky University Medical Center, Tel Aviv, Israel.

### 4.2. Exome Sequencing

ES libraries were prepared using the Illumina DNA PCR-Free Library Prep protocol. Sequencing was conducted on the NovaSeq 6000 (Illumina, San Diego, CA, USA) platform using an S1/S2 reagent kit v1.5 with 150 bp paired-end reads. Reads were aligned to the GRCh37/hg19 reference genome, and resulting BAM files were analyzed using the Integrative Genomics Viewer (IGV) to visually confirm breakpoint regions. Breakpoint coordinates were identified based on split-read and discordant paired-end read patterns. A step-wise variant analysis strategy was performed using the Franklin (version 66) by QIAGEN (formerly Genoox) data analysis software. Automated variant analysis, guided by HPO terms provided in the genetic report, was followed by manual mining by an extended team of bioinformaticians and clinicians familiar with the proband.

### 4.3. Optic Genome Mapping

Ultra-high-molecular-weight (UHMW) DNA was extracted from fresh whole blood following the manufacturer’s protocols (Bionano, San Diego, CA, USA), resuspended in the elution buffer and quantified with a Qubit dsDNA BR Assay Kit (Thermo Fisher Scientific, Waltham, MA, USA). DNA labeling was performed as recommended (Bionano, San Diego, CA, USA). Direct Labeling Enzyme 1 (DLE-1) reactions were carried out using 750 ng of purified UHMW DNA. The fluorescently labeled DNA molecules were imaged sequentially across nanochannels on a Saphyr instrument (Bionano). The OGM procedure reported a DNA molecule N50 (≥20 kb) of 243.38 kb; effective coverage of 430.36; variant calling threshold Inter-Translocation/Intra-Fusion ≥0.05 (or ≥0.02); and a confidence score of 0.21.

### 4.4. In-Gel Analysis of Telomeric Restriction Fragments (TRFs)

Genomic DNA was extracted by a standard proteinase-K–phenol protocol, digested with HinfI restriction endonuclease and separated on 0.6% agarose gel in 1 × TBE, for 1400 V × hr. The gel was dried, denatured, and neutralized as previously described [32]. The dried gel was hybridized simultaneously with a 5′-end-labeled telomeric oligonucleotide, (AACCCT)_3_, and with a combination of 1 kb plus DNA ladder (cat. no. IMDM015; Hy Laboratories, Rehovot, Israel) and a 20 kb DNA fragment ladder (cat. no. SM1541; Thermo Fisher Scientific, Waltham, MA, USA), which were digested with HinfI, dephosphorylated by quick calf intestinal phosphatase (New England Biolabs Inc., Ipswich, MA, USA), and 5′-end-labeled with T4 kinase and [g-32P] ATP. The gel was visualized by Phosphor Imager. Mean TRF length was calculated by the WALTER web tool (https://www.ceitec.eu/chromatin-molecular-complexes/rg51/tab?tabId=125#WALTER, accessed on 11 December 2024) [17] and TeloTool (version 1.3) [18]. The median TRF length was calculated by WALTER.

### 4.5. NanoTelSeq

Genomic DNA was extracted using standard proteinase-K and phenol extraction or by a QIAamp DNA Blood Midi Kit (QIAGEN, Hilden, Germany). Library preparation and sequencing were performed as previously described [16]. Briefly, 5′ phosphorylated, barcoded telorette 3 oligonucleotides (P-5′-TGCTCCGTGCATCTCCAAGGTTAA-BC-CAGCACCTCTAACC-3′, where BC represents one of 24 standard 24 nt-long Nanopore native ligation barcodes (Oxford Nanopore Technologies, Oxford, UK)) were annealed to teltail oligo (5′-TTAACCTTGGAGATGCACGGAGCA-AGCAAT-3′) in NEBuffer 2.1 (New England Biolabs). Pre-annealed telorette (1 or 0.1 µM) was ligated to 0.3–3 µg of genomic DNA samples using 5000 units of T4 DNA ligase (M0202, New England Biolabs, Ipswich, Massachusetts, United States) in 1× T4 ligase buffer in a total volume of 40–50 µL overnight at 30 °C. The ligation reactions were stopped by adding EDTA to a final concentration of 20 μM, and TE buffer was added to the samples to reach a final DNA concentration of 10 ng/µL. The samples were then purified with 0.5× volume of AMPure XP beads (Beckman Coulter Life Sciences, Indianapolis, IN, USA) DNA was quantified by a Qubit dsDNA BR Assay Kit (Invitrogen, Thermo Fisher Scientific, Waltham, MA, USA). Then, 0.2–2 µg from each sample was combined and ligated to 4 μL of the Nanopore sequencing adapter (AMII or NA) and sequenced following the standard protocol for genomic DNA ligation sequencing with native barcoding (SQK-LSK109 with EXP-NBD104 or SQK-NBD114.24, respectively; Oxford Nanopore Technologies). Finally, 0.5–5 μg of the sequencing libraries was loaded onto a Nanopore MinION R9 or PromethION R10 flow cell and run on MinION MK1b or PromethION P2 solo (for SQK-LSK109 or SQK-NBD114.24, respectively).

### 4.6. Computational Processing of the Sequencing Data

Deducing the nucleotide sequence (basecalling) from the raw data (POD5 files) and demultiplexing were performed with Nanopore Dorado software (version 7.11.2) in super accuracy mode. The sequences (FastQ files) were filtered and analyzed by the dedicated application TelomereAnalyzer (https://doi.org/10.5281/zenodo.8379363), searching for the patterns (after reverse complement) of YYAGGG (Y indicates C or T) for R9 chemistry and TTAGGG for R10 chemistry, as described in [33]. Telomeric repeats with 1 mismatch identified TVRs. The telomere length (with and without mismatches) and read length were extracted and presented as plots of telomere density along the entire read (examples shown in Supplementary Figure S1). Telomere density was calculated in a moving window of 100 nt and describes the fraction of telomeric repeats within each 100 nt sequence (from 0—no telomeric sequence to 1—fully telomeric). A telomere is identified by a summed telomere density of at least 2 for at least three consecutive windows, each having a telomere density of at least 0.3. Then, the telomere beginning and end are defined by the first and last telomeric repeat within the identified windows or flanking windows. Telomeric reads were filtered for overall telomere repeat density above 0.75 within the identified telomeric fragment and telomere start within 50 nt from the 5′ end of the read (distal end of the telomere). The minimal read length, median TL and percentage of short telomeres were determined as follows. Telomere reads were initially sorted for read length, from long to short, and a running median for telomere length was calculated from the longest read down to each row. A read length of the running median plus a buffer of 50 nt was chosen as a threshold, and all the shorter reads were excluded. The median of the remaining reads and the percentage of reads with telomeres shorter than 2 kb were calculated. The number of telomeric reads obtained per sample reflects sequencing depth and does not represent a biological replicate; telomere length comparisons between individuals are therefore descriptive, as was the TRF analysis, rather than based on formal statistical hypothesis testing.

## Figures and Tables

**Figure 1:**
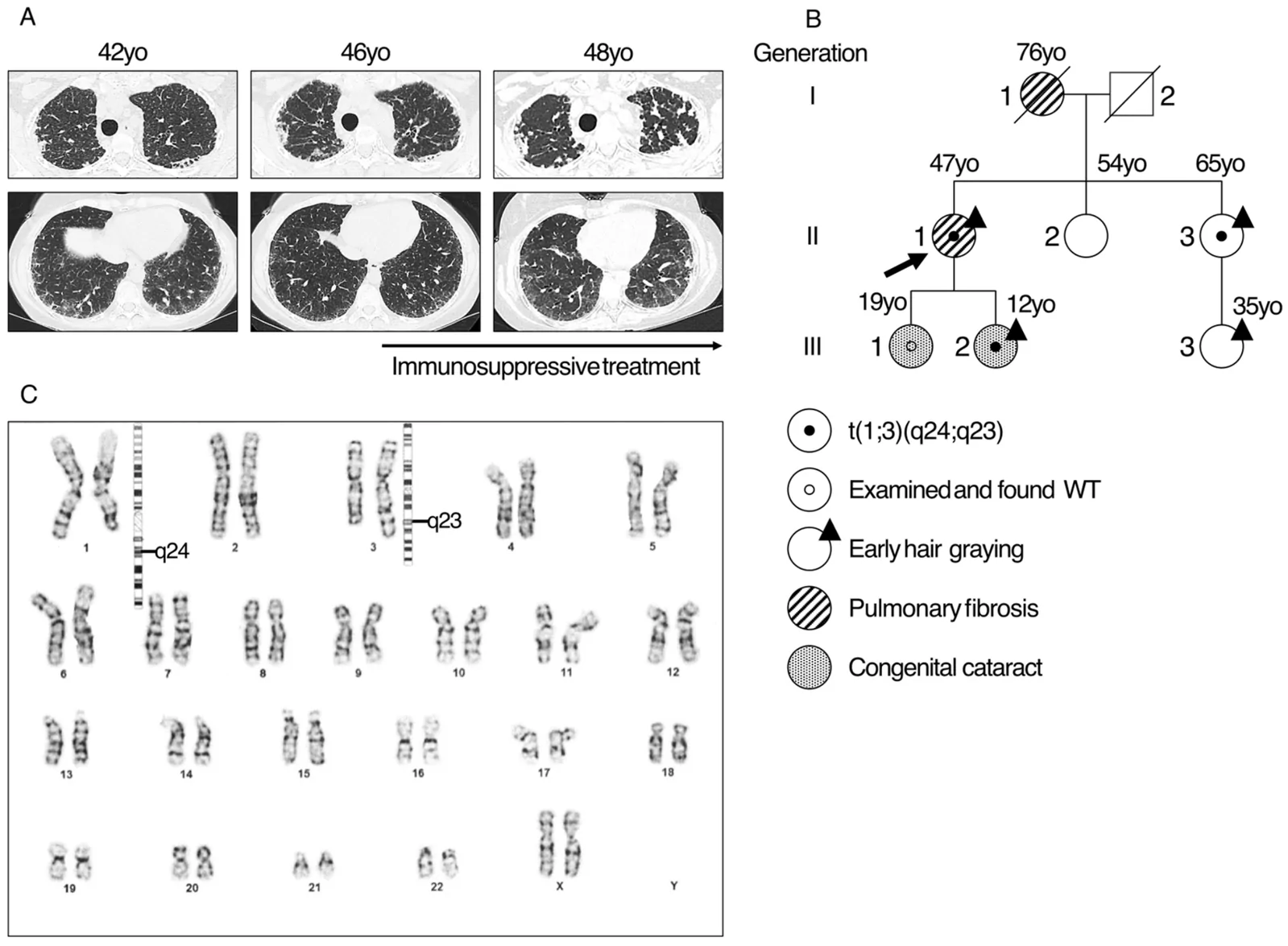
Lung imaging, family history and karyotype. (**A**) Representative CT images of proband’s (II1) lungs at three time points, showing upper lobe subpleural lesions compatible with pleuroparenchymal fibroelastosis (top row) and lower-lobe subpleural reticulations with indeterminate for usual interstitial pneumonia pattern (bottom row). Gradual progression of both upper lobe and lower lobe lesions is observed over time. (**B**) Pedigree of the analyzed family members in three generations (I, II, III): proband (indicated by arrow, II1), her mother (I1), sisters (II2, II3), and two daughters (III1, III2). Crossed symbols represent deceased family members. (**C**) G-banded karyotype of the proband (II1) showing a balanced translocation, 46,XX,t(1;3)(q24;q23), interpreted as benign. The same translocation was identified in the karyotypes of the proband’s sister (II3) and one daughter (III2). The other daughter (III1) tested negative for this familial translocation. Family members I1, II2, and III3 were not tested.

**Figure 2:**
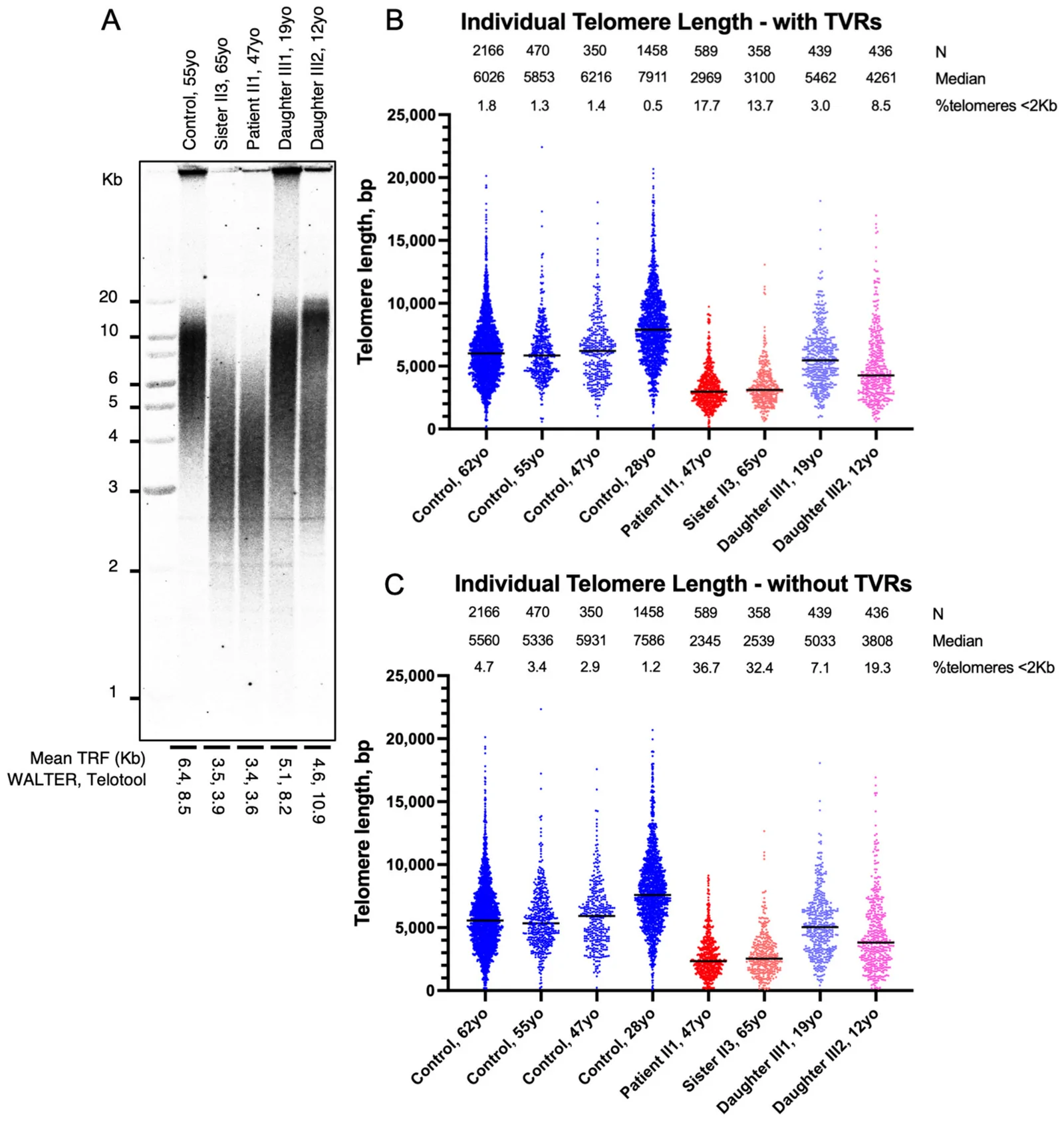
Telomere length by TRFs and NanoTelSeq analyses. (**A**) In-gel hybridization with a telomeric probe to HinfI-digested genomic DNA prepared from blood leukocytes of the proband (II1), her sister (II3), her two daughters (III1, III2), and a healthy 55-year-old female control. Mean TRF length, indicated below the lanes, was calculated by WALTER and TeloTool. (**B**,**C**) Single telomeres were sequenced and measured by NanoTelSeq in blood leukocyte samples from healthy control women at the indicated ages [blue; including the 55 yo sample shown in (**A**)], the proband II1 (red), and family members II3 (light red), III1 (light blue), and III2 (pink). II1, II3 and III2 are carriers of the translocation. TL was determined by the length of the telomere repeat tracts and is presented in scatter plots for each sample. TL in (**B**) includes both canonical (TTAGGG) and telomere variant repeats (TVRs) that vary from the canonical repeat in one position. TL in (**C**) includes only canonical telomeric repeats. The median TL for each sample is indicated by horizontal lines. Number of telomeres, median TL and percentage of telomeres below 2 kb are indicated above.

**Figure 3:**
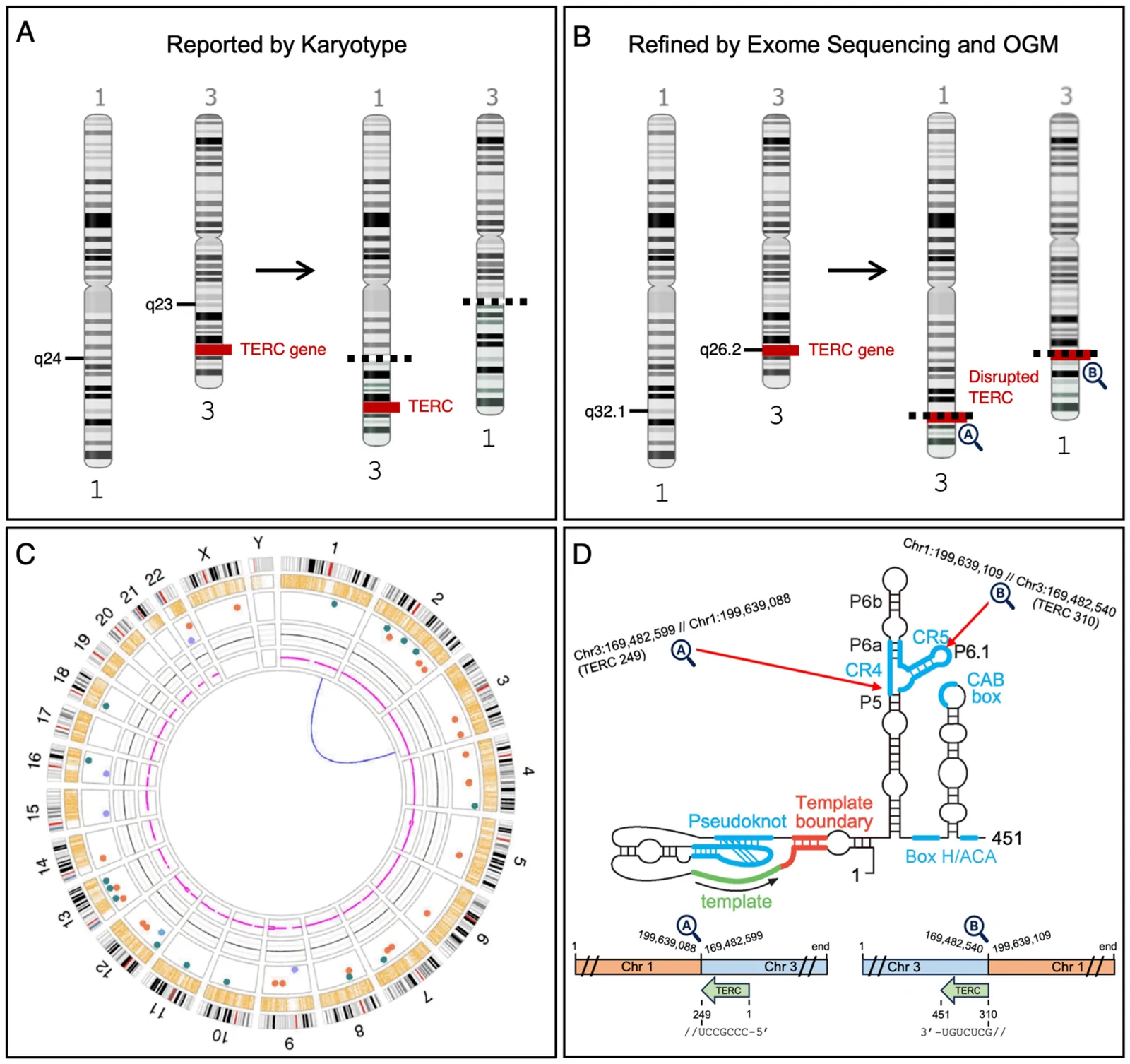
ES and OGM reveal the accurate breakage points of the translocation. (**A**) An illustration of the reciprocal translocation as reported by conventional karyotype [t(1;3)(q24;q23)]. Indicated are the *TERC* locus (red line) and the predicted breakpoints (black dashed lines). (**B**) The refined breakpoints obtained by ES and OGM [t(1;3)(q32.1;q26.2)], resulting in *TERC* disruption. Magnifying glass symbols labeled A and B indicate the breakpoints and are shown at higher resolution in (**D**). The G-banded chromosome illustrations were taken from the NIH Genome Data Browser (https://www.ncbi.nlm.nih.gov/gdv/browser/genome/?id=GCF_000001405.40, accessed on 1 May 2025). (**C**) OGM results shown by Circos plot reveal a fusion line between chromosomes 1 and 3 (DNA molecule N50 (≥20 kbp) 243.38 kbp; effective coverage 430.36; variant calling threshold inter-translocation/intra-fusion: ≥0.05 (or ≥0.02); confidence score 0.21). (**D**) Upper illustration shows the secondary structure of the 451 nt *TERC* RNA, indicating the template for telomeric repeat synthesis (green) and other essential and conserved domains (red and magenta). Red arrows show the two ends of the breakpoint in chromosome 3, which fused to the indicated broken ends of chromosome 1, disrupting *TERC* in the CR4-CR5 domain. The *TERC* sequence between 250 and 309 is missing from the two parts. Bottom schemes show the two chromosomes affected by the translocation. Chromosome 1 (left) fused to the translocated fragment of chromosome 3, and chromosome 3 (right) fused to the translocated fragment of chromosome 1. Indicated on the schemes are the two chromosomes’ fragments, the genomic coordinates on both sides of the fusion points, the disrupted *TERC*, and the *TERC* sequences at the fusion points. *TERC* is encoded by the reverse-complement strand.

**Figure 4:**
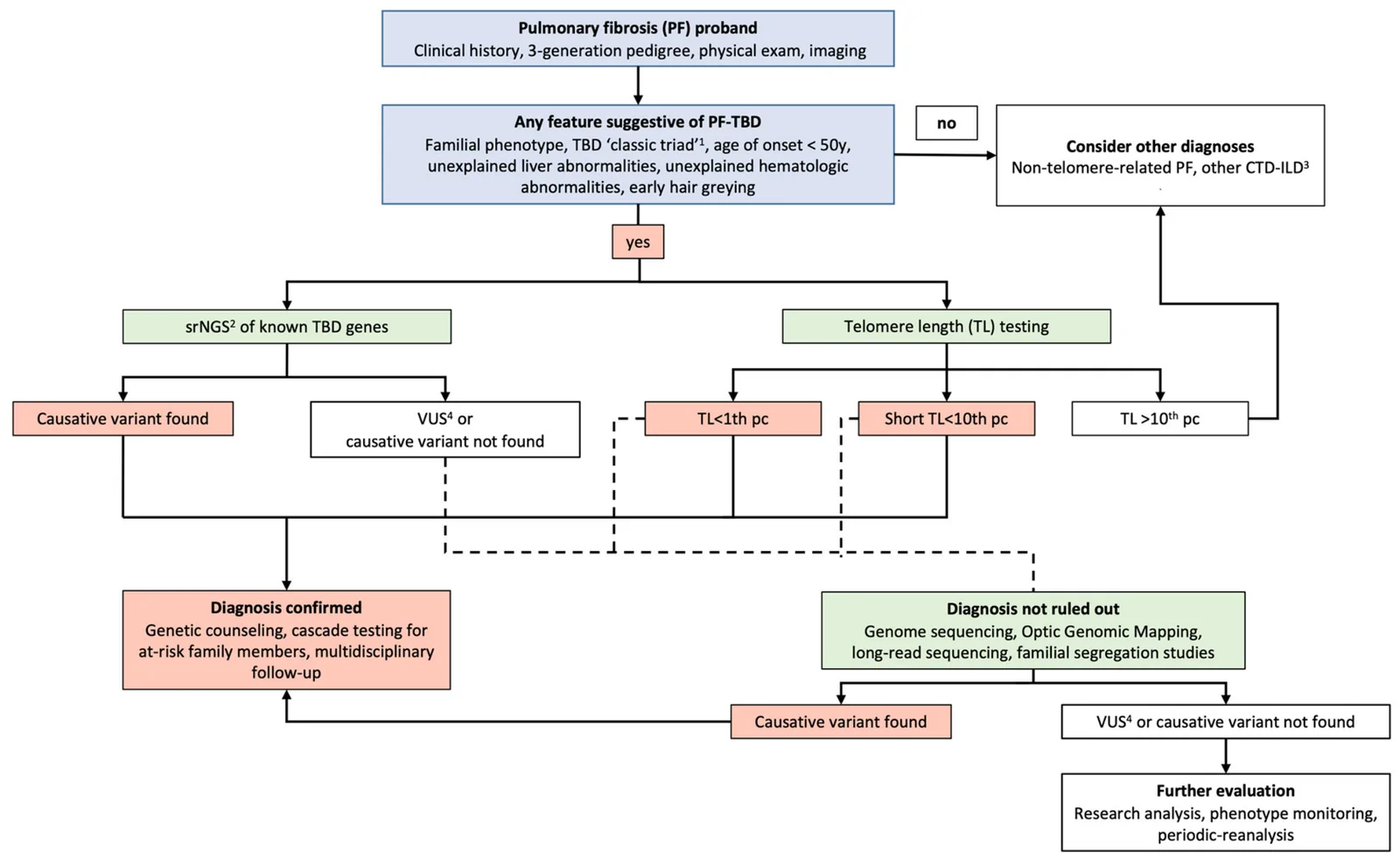
Diagnostic workflow for suspected TBD-PF patients. The initial screening step is based on the clinical history and features outlined above. We propose that patients with a clinical phenotype consistent with PF-TBD undergo both genetic sequencing and TL testing early in the diagnostic evaluation rather than following a sequential approach in which TL testing is performed first and genetic sequencing is pursued subsequently (or vice versa). Performing both assessments early in the diagnostic pathway may reduce interpretative ambiguity and minimize the risk of false-negative results associated with either test alone. The TL percentiles indicated in the flowchart refer to measurements obtained by flow-FISH, which remains the common method for clinical TL assessment. However, in the future, as NanoTelSeq becomes validated and accredited as a clinical test, it will provide a more accurate and clinically informative evaluation of median TL and the percentage of critically short telomeres. Blue boxes represent clinical features highly suggestive of PF-TBD; green boxes, laboratory tests; light red boxes, indicative findings. Lines indicate the flow for conclusive results and dashed lines describe the flow for inconclusive results. ^1^ Skin pigmentation, nail dystrophy, oral leukoplakia; ^2^ short-read next-generation sequencing; ^3^ connective-tissue disease–interstitial lung disease; ^4^ variant of unknown significance.

**Table 1:** Genetic studies, telomere length testing and clinical features.

				Telomeric Restriction Fragments (TRF)			NanoTelSeq (With TVRs)		NanoTelSeq (Without TVRs)		
				Telo Tool	WALTER						
ID	Relationship to Proband	Translocation Carrier	Tests Performed	Mean TRF (kb)	Mean TRF (kb)	Median (kb)	Median (kb)	Telomeres <2 kb (%)	Median (kb)	LTL <2 kb (%)	Clinical Manifestations
Control, 62 yo	Unrelated control (62 year-old female)	N/A	NanoTelSeq	N/A	N/A	N/A	6.03	1.8	5.56	4.7	N/A
Control, 55 yo	Unrelated control (55 year-old female)	N/A	TRF, NanoTelSeq	8.5	6.4	6.2	5.85	1.3	5.34	3.4	N/A
Control, 47 yo	Unrelated control (47 year-old female)	N/A	NanoTelSeq	N/A	N/A	N/A	6.22	1.4	5.93	2.9	N/A
Control, 28 yo	Unrelated control (28 year-old female)	N/A	NanoTelSeq	N/A	N/A	N/A	7.91	0.5	7.59	1.2	N/A
II1	Proband	Yes	Karyotype, ES, OGM, TRF, NanoTelSeq	3.6	3.4	3.2	2.97	17.7	2.35	36.7	Microcephaly, premature hair graying (age 11), frontal fibrosing alopecia, osteoporosis
II3	Sister	Yes	Karyotype, ES, TRF, NanoTelSeq	3.9	3.5	3.3	3.10	13.7	2.54	32.4	Premature hair graying
III1	Daughter (elder)	No	Karyotype, ES, TRF, NanoTelSeq	8.2	5.1	4.9	5.46	3.0	5.03	7.1	Bilateral congenital cataracts
III2	Daughter (younger)	Yes	Karyotype (prenatal), ES, TRF, NanoTelSeq	10.9	4.6	4.2	4.26	8.5	3.81	19.3	Microcephaly, early hair graying (age 9), VSD, bilateral congenital cataracts

TRF, telomeric restriction fragment; TVR, telomere variant repeat; ES, exome sequencing; kb, kilobase. NanoTelSeq values are shown with and without inclusion of TVRs. I1 (proband’s mother, deceased), II2 and III3 (proband’s sister and niece) were not tested for telomere length and are therefore not included in this table.

## Data Availability

The deidentified datasets generated and analyzed during the current study are available from the corresponding authors upon request.

## Supplementary Materials

The following supporting information can be downloaded at https://www.mdpi.com/article/10.3390/ijms27198667/s1.
