# Supplementary material for: A Balanced Chromosomal Rearrangement Disrupting the Telomerase RNA Component Gene Causes Short Telomere-Mediated Pulmonary Fibrosis
Source: Int J Mol Sci. 2026 Sep 28;27(19):8667. doi: 10.3390/ijms27198667 (PMC13665144; doi:10.3390/ijms27198667)
Supplement: Supplementary file 1 [file ijms-27-08667-s001.zip › ijms-4509685-supplementary.pdf]

Supplementary Material

Clinical course of pulmonary disease

The proband first presented to a pulmonologist due to effort dyspnea at the age of 40 years old. She had smoked 1-2 cigarettes per day from age 16 to 40 years, and worked in an office with no relevant occupational exposures. Her past medical history included arthritis of unknown cause for eight months at age 25 with spontaneous remission. Her mother died from idiopathic pulmonary fibrosis (IPF) at age 76.

Her physical examination revealed clubbing, minimal bibasilar "velcro" rhales, no signs of arthritis sclerodactyly or mechanic hands, and normal O_2 saturation at rest. Forced vital capacity (FVC) at presentation was mildly reduced at 65% predicted, and diffusion capacity for carbon monoxide (DLCO) was already severely reduced at 39%. At presentation, chest computed tomography (CT) revealed a pleuroparenchymal fibroelastosis (PPFE) pattern in upper lung zones and an indeterminate usual interstitial pneumonia (UIP) pattern in lower lung zones. A diagnostic bronchoscopy showed a non-diagnostic pattern of mild fibrosis in septae and a few germinal centers. Bronchoalveolar lavage revealed 3% neutrophils, 12% eosinophils, 7% lymphocytes, and 77% macrophages with a CD4 to CD8 ratio of 0.7.

She was given a working diagnosis of IPF and received nintedanib anti-fibrotic treatment, which was switched to pirfenidone after four years because of side effects. However, her respiratory condition gradually deteriorated, reflected by progression of PF on CT (Figure 1A) and worsening lung function tests. Seven years after presentation, her diagnosis was re-evaluated by multidisciplinary discussion in light of her past arthritis, elevated ESR and CRP, germinal centers in lung biopsy, and a significant exposure to mold and birds. Of note, nailfold capillaroscopy and broad autoimmune serologies, including scleroderma and myositis panels, were negative. She did not exhibit other symptoms suggestive of a connective-tissue disorder. A provisional diagnosis of fibrotic hypersensitivity pneumonitis (fHP) was made, and prednisone 40 mg was started with gradual tapering down, but she continued to deteriorate. Within a year she was enlisted for lung transplantation. By age 49 she was oxygen dependent 24 hrs/day, her FVC was 28%, she was unable to complete DLCO measurement, and her 6-minute walking distance was 278 meters on oxygen (compared to 480 meters on room air three years prior). Nine years after presentation, she underwent a successful lung transplantation.

Explant pathology showed interstitial fibrosis that was most prominent peripherally; however, there were areas of peripheral sparing, peribronchiolar fibrosis, patchy moderate chronic inflammation and a rare possible non-necrotizing granuloma, that are compatible with fHP.

Her post-transplant drug regimen included prednisone 10 mg/day, tacrolimus (target trough level 10-12 ng/mL), and mycophenolate mofetil 750 mg bi-daily, that was switched to azathioprine 50 mg/day due to gastrointestinal discomfort. An episode of acute rejection two months post-transplant was treated with high dose steroids, which stabilized her condition. Currently, 1.5 years post-transplantation, she is doing well with near-normal lung function tests (FEV1 76-82%, FVC 77-81%, FEV1/FVC 0.85).

List of genes included in the panel analysis of pulmonary fibrosis and telomere biology disease genes

ABC3, ABCA3, ABCC7, ACD, AFF4, AP3B1, APT1, APT1LG1, AS, ATP11A, BACH2, BCH, BLM, BMP15, BNC, BNC1, BTNL2, C17orf62, C17orf68, C20orf41, C8orf38, CASP10, CAV, CAV1, CBP1, CBP2, CCN2, CCR6, CDC4L, CDON, CELIAC3, CF, CFTR, CLN2, CSPP1, CTC1, CTCF, CTGF, CTLA4, Cxorf3, CYBC1, DISP1, DKC, DKC1, DLL1, DPD1, DPP9, DSP, EBS1, ECHD, EHHADH, FAM111B, FAM13A1, FAS, FAS1, FASLG, FCG2, FCGR2, FCGR2A, FCGR2A1, FGF8, FGFR1, FKH1.15, FLT2, FOXE1, FOXE2, FOXH1, FSHR, FTZF1, GAS1, GATM, GBA, GBA1, GLI2, GLUC, HAPB2, HLA-B, HLA-DP1A, HLA-DPB1, HLA-DPA1, HLA-DR1B, HLA-DR1B1, HLP3, HPE2, HPE3, HPE4, HPS, HPS1, HPS4, IDDM12, IFI41, IFI75, IL1RN, IRF5, ITCH, KAL2, KIAA04146, KIAA0319L, KIAA0586, LAT, LRBA, LRR6C6, MARS, MARS1, MDM4, MMP1, MRPS22, MRX12, MRX35, MUC5, MUC5B, NAF1, NBCCS1, NDUFAF6, NHLRC2, NHP2, NKX2-1, NKX2A, NOLA1, NOLA2, NOLA3, NOP10, NPC2, NPT2, NR5A1, NUP107, OBFC1, ODG1, PARN, PEPD, PIGY, PLCH1, PLCL3, PLEC, PLEC1, POLR3H, POT1, PRIM1, PRKCD, PRTN3, PSMC3IP, PTCH, PTCH1, PTPN22, PTPN8, RAC2, RASGRP1, RCBTB1, RETL1, RIPK1, RNF168, RPA1, RTEL1, SCAR7, SERPINH1, SERPINH2, SFTPA1, SFTPA2, SFTPB, SFTPC, SHH, SLI1, SLX3, SLC17A2, SLC34A1, SLC34A2, SMC1A, SMC1L1, SPTB, STAG2, STIL, STING1, STN1, STRL22, IDGF1, TERC, TERT, TGFB1, TGIF1, THOC2, TIN2F, TIT1, TITF2, TMEM173, TNFRSF16, TNFSF6, TOM1, TP53, TPP1, TS, TYMS, WARP53, WDR79, WRAP53, ZCCHC8, ZIC2, ZNF699, ZSWIM7.

Supplementary Figure S1: Telomeric sequences captured by NanoTelSeq

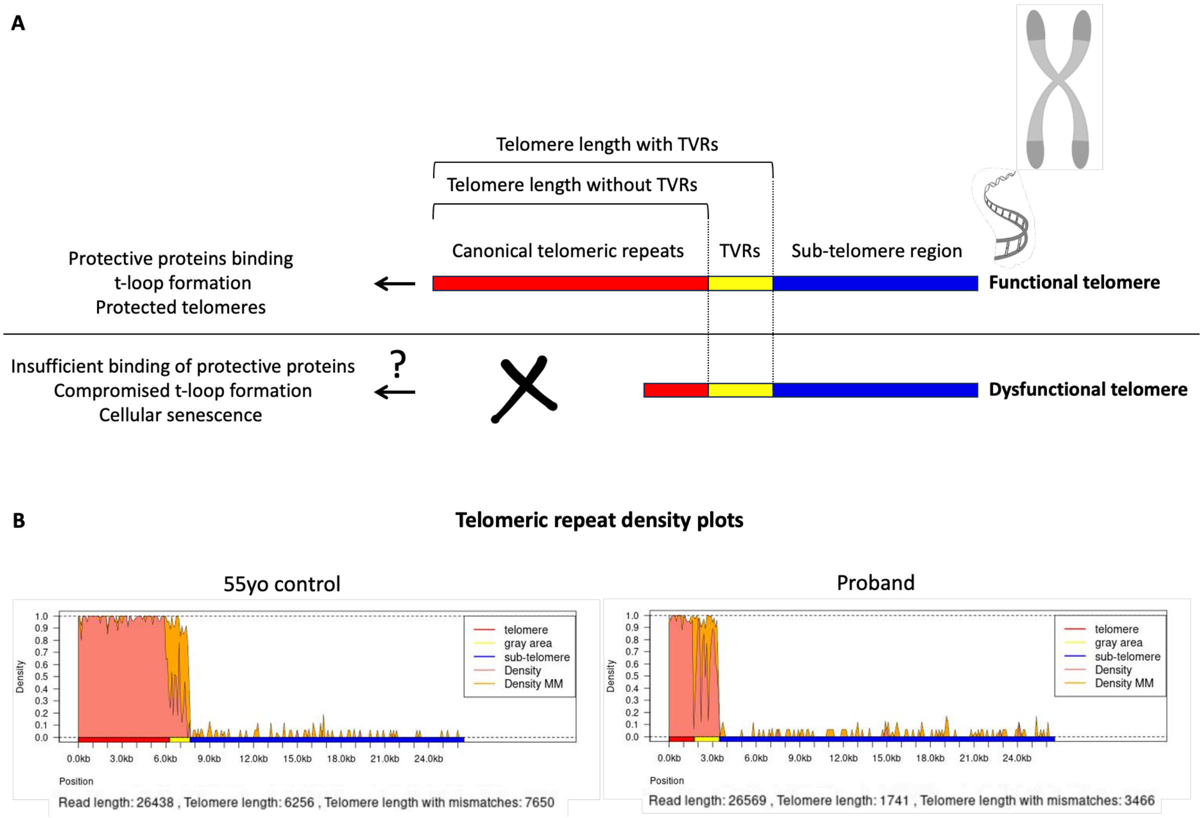

Supplementary Figure S1. Telomeric sequences captured by NanoTelSeq

(A) Schematic illustration of telomeric sequences captured by NanoTelSeq and their potential implications in telomere biology disorders (TBDs). Telomere variant repeats (TVRs) are present in some telomeres, between the subtelomere and the more distal canonical telomeric repeats. While the length of the TVR region appears comparable between healthy individuals and TBD patients, the region of canonical telomeric repeats is shorter. While canonical repeats are the primary determinants of telomere function, TVR repeats may have reduced affinity to protective telomere binding proteins such as shelterin and compromised ability to form the protective t-loop structure. When canonical repeats are fewer than TVRs, telomere function may be compromised, leading to loss of telomere protection, triggering DNA damage response and cellular senescence. (B) Graphs of single telomeres, plotting the telomeric repeat density (Y axis - from 0 to 1) along the length of the read in a moving window of 100 bp (x axis). Canonical telomeric repeats are shown in red, while TVRs (repeats with one mismatch), in orange. Subtelomeric region is indicated in blue, regions with canonical telomeric repeats density >0.6 in red, and regions with both canonical and TVRs repeat density >0.6 in yellow. The upper panel shows a representative telomere from a 55-year-old control, illustrating a relatively long telomere when including or excluding TVRs (7.7 kb and 6.3 kb, respectively). The lower panel shows a representative telomere from the proband, with a length of 3.5 kb when including TVRs (above the critical threshold of 2 kb), but 1.7 kb when excluding TVRs.

Supplementary Figure S2: Exome sequencing of the proband

A

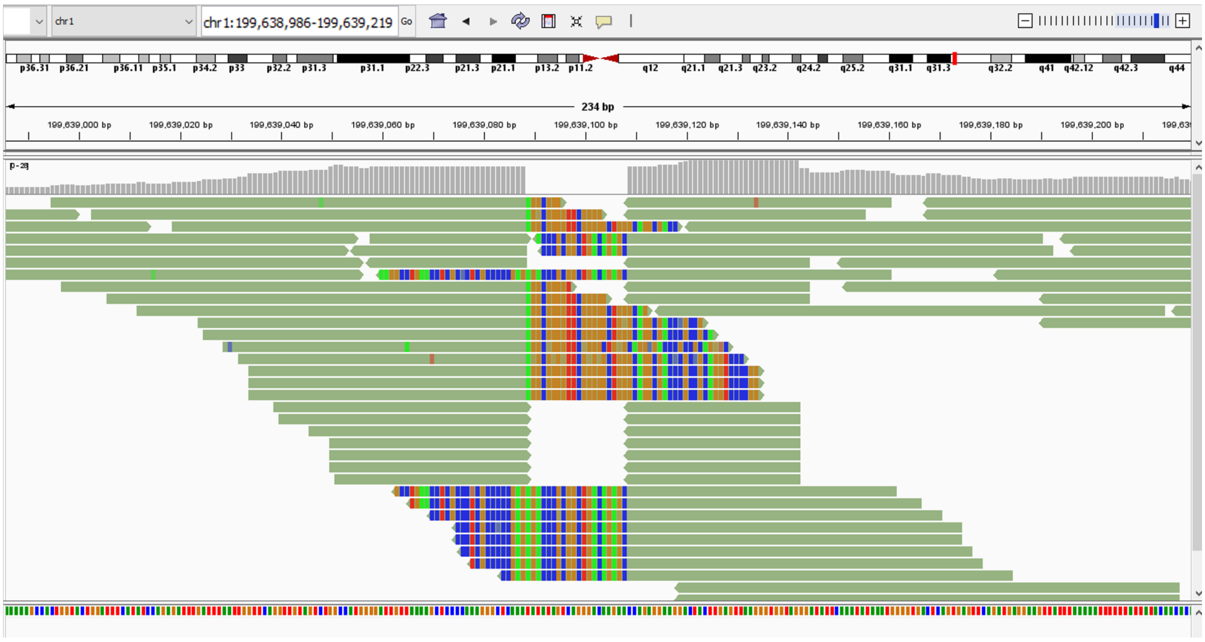

B

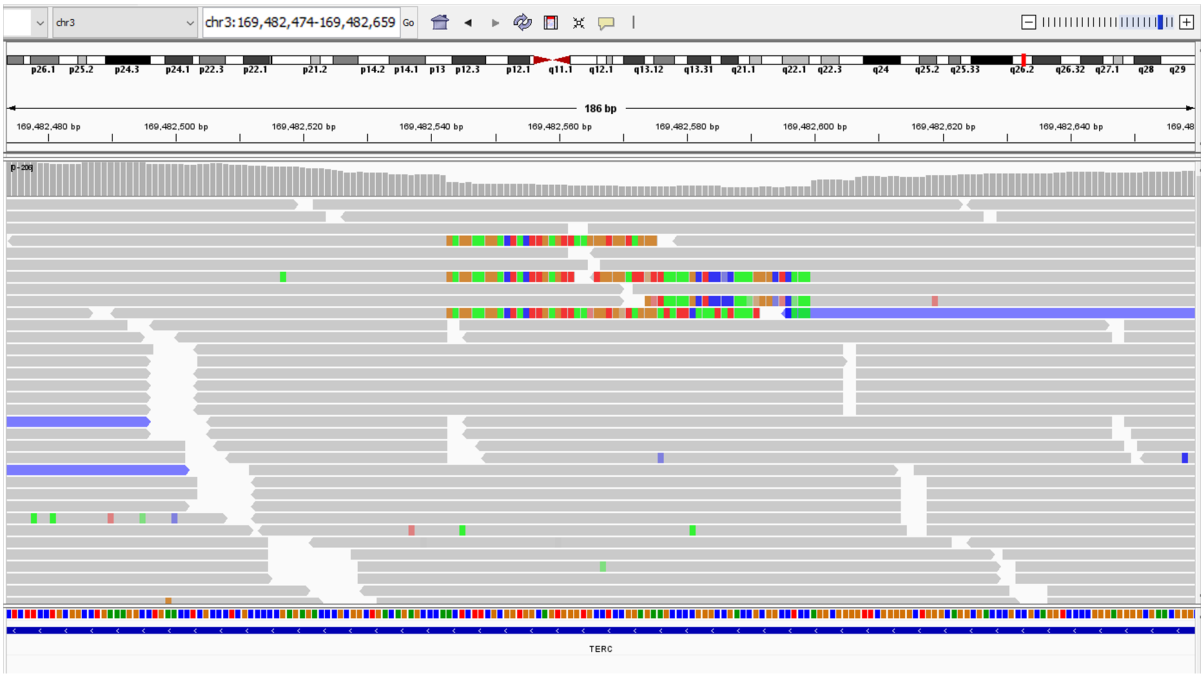

Supplementary Figure S2: Exome sequencing of the proband

Integrated Genomics Viewer (IGV) visualization of sequencing reads of the proband aligned to the human reference genome (GRCh37/hg19). (A) The sequence segments shown in green align to chromosome 1 while the colored sequences represent misaligned segments corresponding to the *TERC* gene on chromosome 3, identifying the breakpoint within chromosome 1 (Chr1: 199,639,088–199,639,109). (B) The sequence segments shown in grey align to chromosome 3 while the colored sequences represent misaligned segments corresponding to the breakpoint locus on chromosome 1, identifying the breakpoint within the *TERC* gene on chromosome 3 (Chr3: 169,482,540–169,482,599) (see Figure 3D in the main text). Translocation breakage points are indicated by red line over the chromosome illustration above.

*The nomenclature for the translocation (t(1;3)(q32.1;q26.2)):
NC_000001.10:g.199639109_qterdelins[NC_000003.11:g.169482542_qter]
NC_000003.11:g.169482607_qterdelins[NC_000001.10:g.199639089_qter]

Supplementary figure S3: Exome sequencing of the proband's sister and daughters

A

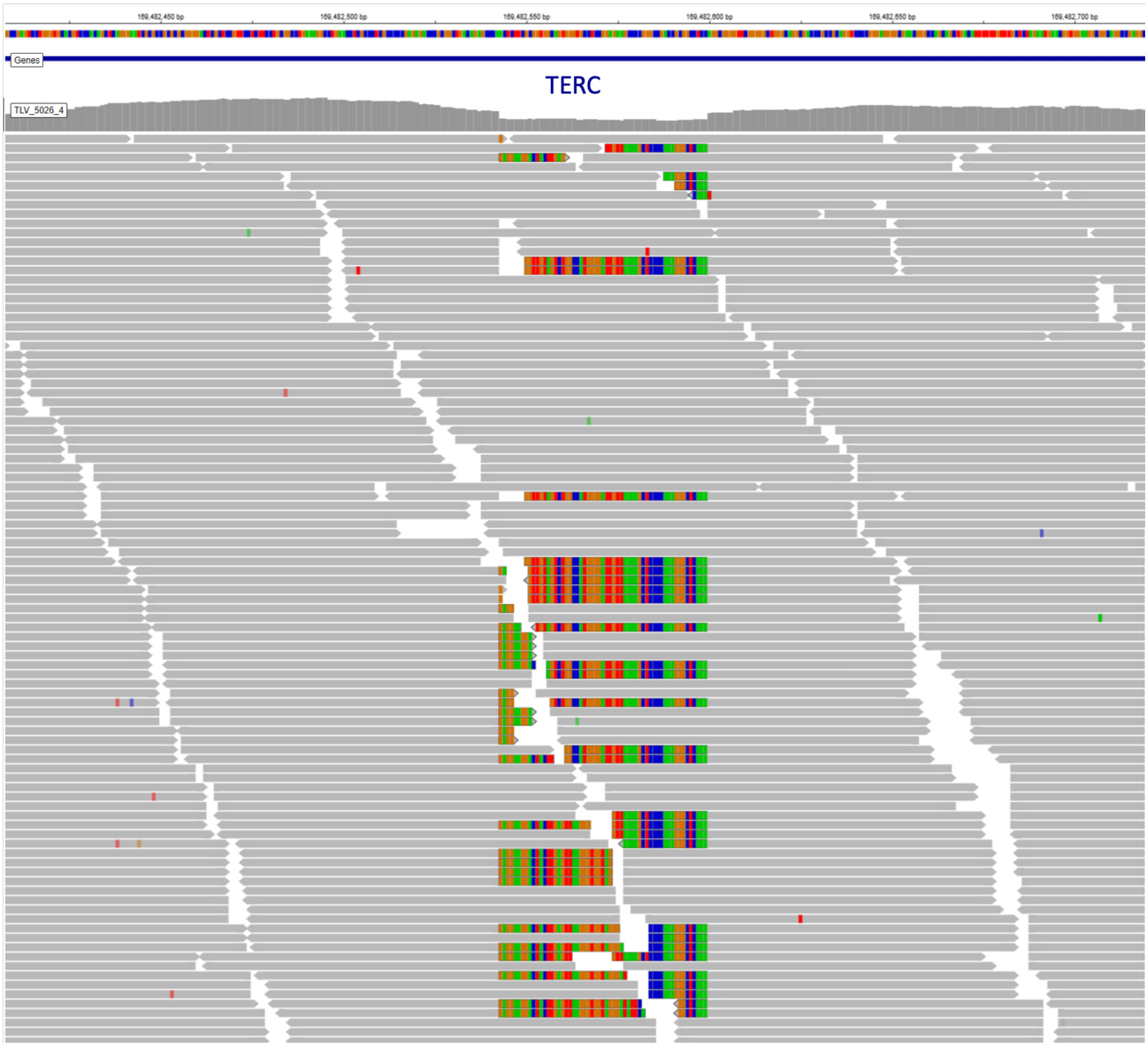

B

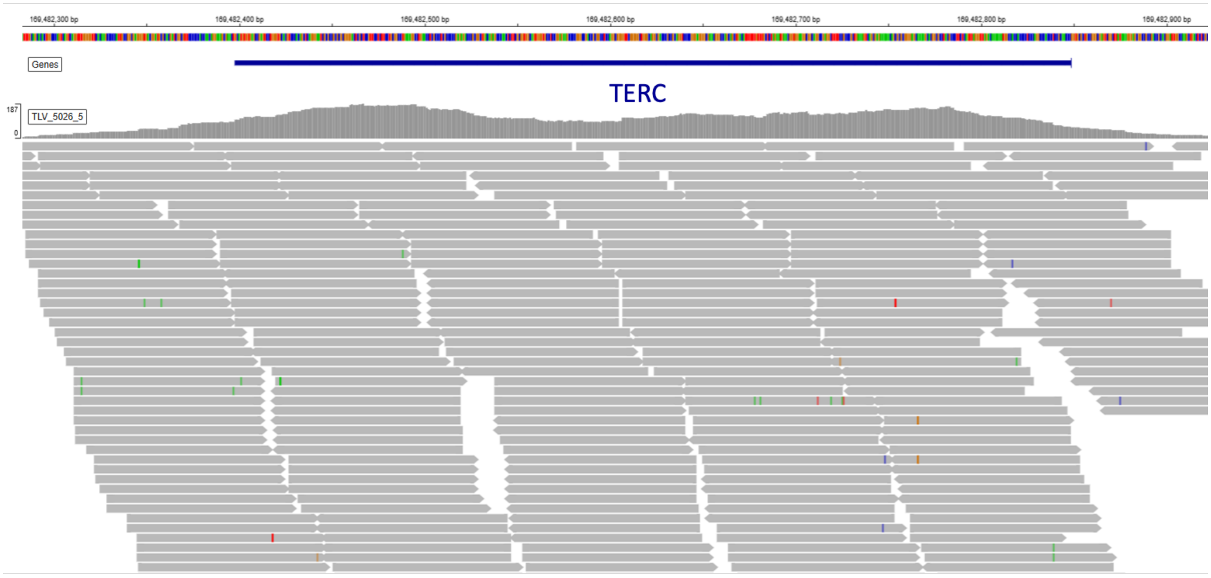

C

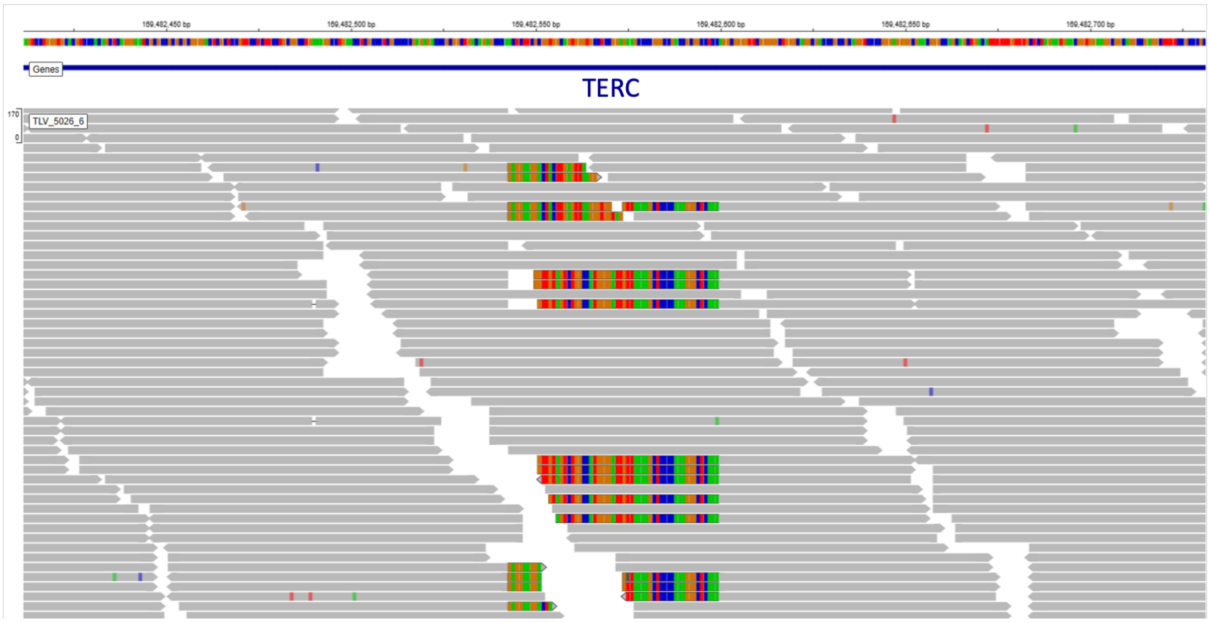

<u>Supplementary figure S3: Exome sequencing of the proband's sister and daughters</u>

Integrated Genomics Viewer (IGV) visualization of exome sequencing reads (grey) aligned to the human reference genome chromosome 1. The colored segments represent misaligned *TERC* sequences in the proband’s sister II3 (A) and younger daughter III2 (C), but not in the elder daughter III1 (B).

Supplementary Figure S4: OGM variant details

Filtered Structural Variant

SMAP ID: 985

Variant Details

Type	translocation_interchr
Location	chr3:199,638,595 ; chr1:169,487,747
Confidence	0.3
Present % Control Samples	0
Nearest Non-overlap Gene	ACTRT3
Found in Self Molecules	yes
Overlapping Genes	-
Overlapping Features	-
Zygosity	heterozygous
Overlapping Genes Count	0
Present in % of Bng Control Samples With The Same Enzyme	0
Fail Assembly Chimeric Score	pass
Putative Gene Fusion	
Molecule Count	26
Number of Overlap Dgv Calls	-1
UCSC Web Link 1	UCSC Web Link 1
UCSC Web Link 2	UCSC Web Link 2

Copy Number Variants

No Overlapping Copy Number variants found.

Supplementary figure S4: OGM variant details

The translocation details obtained from OGM, including the accurate breakage points of the translocation.

Supplemetary Table S1. Comparative analysis of structural variation detection methodologies

Technology	Advantages	Limitations
**Karyotyping**	• **Gold standard** for global structural variants<br><br>• Directly visualizes reciprocal & Robertsonian balanced translocations<br><br>• Fully positional (identifies participating chromosomes)	• Low resolution limit (~5–10 Mb)<br><br>• Requires live cell culture/metaphase spreads<br><br>• Misses micro-translocations and cryptic rearrangements
**Chromosomal Microarray**	• High resolution for sub-microscopic copy-number changes	• **Blind to balanced translocations** (no loss or gain of genetic material occurs)
**Short-Read Exome Sequencing**	• Captures coding SNVs/Indels and small CNVs at gene-level precision	• **Incapable** of detecting balanced translocations; short reads (~150 bp) fail to span non-coding, repetitive breakpoints
**Optical Genome Mapping**	• High-throughput genome-wide reconstruction on ultra-high-molecular-weight DNA<br><br>• Reliably detects balanced translocations at ~10 kb resolution without cell culture	• Does not provide base-pair sequence resolution at breakpoints<br><br>• Requires specialized high-molecular-weight DNA extraction protocols
**Manual Split-Read Review** (BAM inspection of srGS/srES)	• Resolves exact nucleotide-level breakpoints if a read pair crosses the junction	• Highly reliant on reads mapping precisely across junctions; fails in repetitive/low-complexity region breakpoints<br><br>• Labor-intensive and low throughput
**Long-Read Sequencing** (PacBio or Nanopore)	• Captures the precise breakpoints and sequence composition of structural variants	• Requires specialized high-molecular-weight DNA extraction protocols<br><br>• Labor-intensive and expensive
